# THE ROLE OF POLICY CONTEXT IN INDIVIDUAL LONG-TERM CARE DECISIONS IN OECD COUNTRIES

**DOI:** 10.1093/geroni/igz038.557

**Published:** 2019-11-08

**Authors:** Selena Caldera

**Affiliations:** LBJ School of Public Affairs - University of Texas at Austin, Austin, Texas, United States

## Abstract

While family caregiving of the elderly has long been part of the cultural life of most OECD countries, longer life expectancy combined with low fertility rates has increased the share of the population dependent on current workers and minimized the available population of informal caregivers. The demand for expanded public provision of long-term care (LTC) resulting from this demographic shift prompted reforms in many OECD countries in the 1990s and 2000s. Differences in these reforms provide an opportunity to examine how individual choices between formal and informal care types are shaped by the policy context. I use longitudinal data on elders in three OECD countries, Sweden, Germany, and Japan, to examine LTC decisions under three varied approaches to population aging. The direction of LTC reforms in each country has been shaped by the existing model of care provision and financial constraints. In response to cost pressures, Sweden introduced need-based provision, financial devolution, and market-based approaches to its universal care model. Germany and Japan, in contrast, widely expanded restricted LTC coverage through public LTC insurance models. I use three multinomial logistic models of the LTC decision to test how differing policy schemes influence choices between formal and informal care. Using longitudinal Global Gateway to Aging data for each country, I model the LTC decision in each country as a factor of demographic and need characteristics of the elder experiencing limitations, characteristics of their family, and eligibility for publicly-provided LTC.

